# Opening the Implicit Leadership Theories’ Black Box: An Experimental Approach with Conjoint Analysis

**DOI:** 10.3389/fpsyg.2018.00100

**Published:** 2018-02-07

**Authors:** Gustavo M. Tavares, Filipe Sobral, Rafael Goldszmidt, Felipe Araújo

**Affiliations:** Center for Behavioral Research, Brazilian School of Public and Business Administration, Rio de Janeiro, Brazil

**Keywords:** implicit leadership theories, leadership perceptions, leadership prototypes, leadership categorization, conjoint analysis

## Abstract

Although research on implicit leadership theories (ILTs) has concentrated on determining which attributes define a leadership prototype, little attention has been paid to testing the relative importance of each of these attributes for individuals’ leadership perceptions. Building on socio-cognitive theories of impression processes, we experimentally explore the formation of leadership perceptions based on the recognition of six key attributes in a series of three experimental studies comprising 566 US-based participants recruited online via Amazon Mechanical Turk. Our results show that while certain attributes play an important role in the leader categorization process, others are less relevant. We also demonstrate that some attributes’ importance is contingent on the presence of other attributes and on the leadership schema type activated in respondents’ minds. Consistent with the Leadership Categorization Theory, our findings support the premise that individuals cognitively hold a superordinate leadership prototype, which imposes constraints on their more basic level prototypes. We discuss the implications of these results for leadership theory and practice.

## Introduction

When do we recognize someone as a leader? Socio-cognitive and information processing approaches to leadership (e.g., Lord and Maher, 1991) answer this question based on the notion of implicit leadership theories (ILTs). ILTs are defined as cognitive structures or prototypes constituted by individuals’ conceptions of the traits and behaviors that characterize a leader (Epitropaki et al., 2013). ILTs’ relevance to leadership relies on the premise that followers use these prototypes as a benchmark to categorize others as leaders (Junker and van Dick, 2014). This categorization process influence followers’ attitudes and behaviors toward leaders (Epitropaki and Martin, 2005). When a leader is closer to a follower’s idealized image of a leader (i.e., the higher a leader’s prototypicality), that leader will be evaluated more positively (Foti et al., 2017).

Research has shown that the overall congruence between followers’ ILTs and the recognized attributes in leaders is ultimately related to organizational outcomes, such as leader-member exchange (LMX) quality, followers’ identification with the leader, organizational commitment, job satisfaction, and well-being (e.g., Epitropaki and Martin, 2005; Topakas, 2011; Van Quaquebeke et al., 2014). However, because these studies focused on the effects of leader prototypicality *as a whole*, they say little about the importance of each individual leader attribute in the leadership categorization process. Indeed, congruence models (e.g., Epitropaki and Martin, 2005) have implicitly assumed that all leader attributes are equally meaningful in predicting outcomes (Foti et al., 2017). As such, we cannot know the extent to which each prototypical attribute individually contributed to those outcomes or which prototypical attributes are most relevant for recognizing someone as a leader.

Because the leadership prototype is comprised of a set of attributes followers expect leaders to have—such as intelligence, sensitivity, dedication, and dynamism (Epitropaki and Martin, 2004)—someone can be perceived as being close to the prototype in some dimensions (e.g., sensitivity), but distant from the prototype in others (e.g., intelligence). However, we argue that different combinations of attributes’ levels (e.g., an intelligent but insensitive leader vs. a sensitive but unintelligent leader) may lead to different outcomes in terms of leadership perceptions. This result is likely to occur because the information about each of a leader’s attributes may receive distinct weights when cognitively integrated by followers to form an overall impression of that leader (e.g., Anderson, 2008).

For example, suppose that on a scale from 1 (low) to 9 (high), an individual expects leaders to be an 8 in terms of both sensitivity and intelligence. Now assume that this individual is forming an impression of two leaders: the first is perceived as being an 8 in sensitivity and a 4 in intelligence, while the second one is a 4 in sensitivity and an 8 in intelligence. Numerically, both leaders are, on average, equally distant from the prototype. However, maintaining all other attributes constant, would these leaders be categorized equally in terms of leadership prototypicality? According to the congruence models used by previous research, the answer is *yes*. However, if we assume that some leader attributes carry more weight than others in the categorization process, the answer to this question is *no*.

We argue that the formation of leadership perceptions results from a *cognitive algebra* (see Anderson, 1996, 2008), in which the information about leader traits receives distinct weights depending on its contingent relevance. However, we believe this process is not purely additive. Instead, we argue that followers’ impression formation of a leader follows Asch’s (1946) configural model of perception, in which individual attributes’ meanings are contingent on the presence of other attributes. This more dynamic view of the impression process is consistent with the holistic approach to impression formation (see Baumeister and Finkel, 2010; Fiske and Taylor, 2013). Furthermore, consistent with the connectionist approach to leadership categorization (e.g., Lord and Shondrick, 2011), we consider that the way followers integrate the information regarding their leaders’ characteristics is influenced by external factors.

Although ILTs studies have allowed researchers to identify several leader attributes with high prototypicality, no effort has been made so far to investigate experimentally the extent to which the recognition of each of these attributes affects individuals’ leadership perceptions. To fill this gap, we draw on socio-cognitive theories on impression processes and leadership categorization to experimentally explore the formation of leadership perceptions based on the recognition of prototypical attributes. To do so, we use conjoint analysis (CA), an experimental technique that allows a large set of variables to be manipulated and the relative importance of an object’s attributes to be measured (Rao, 2014). Specifically, by using CA, we could test the causal link between the recognition of each attribute and leadership perceptions.

Through our manipulations, we make four primary contributions to the ILTs literature. First, we show that the importance of the (anti)prototypical attributes proposed by Epitropaki and Martin (2005) for individuals’ recognition-based leadership perceptions is heterogeneous. Second, building on a more holistic approach to impression processes (see Fiske and Taylor, 2013), we demonstrate the configural nature of leadership perception formation, i.e., we explore if the presence of a specific attribute can enhance or weaken other attributes’ effects. Third, we show both the dynamism and the consistency of individuals’ leadership schemas after manipulating the leadership context (e.g., military, business, and political), thus empirically supporting the theoretical propositions of the connectionist approach to leadership categorization (e.g., Brown and Lord, 2001). Finally, we present the advantages of using CA in ILTs research.

## ILTs and Leadership Categorization

Implicit Leadership Theories were first proposed by Eden and Leviatan (1975) based on the notion of implicit theories of personality. Eden and Leviatan asked students to rate leadership behaviors in a hypothetical situation. After conducting a factor analysis, the four resulting factors (support, work facilitation, interaction facilitation, and goal emphasis) were the same as those found in prior studies in which individuals rated their actual organizations’ leaders (Halpin and Winer, 1957). This finding indicated that a connection among leadership attributes was already in the participants’ minds, independently of whom they were evaluating.

Lord et al. (1984) advanced ILTs research using Categorization Theory principles (Rosch, 1978) to propose the existence of a leadership prototype. According to Rosch (1978), a prototype is an abstract composite of the most representative characteristics of a category’s members. Thus, the leadership prototype can be defined as an abstract cognitive structure formed by the attributes that are the most associated with leaders (Epitropaki et al., 2013). Accordingly, individuals are categorized as leaders when their characteristics match with those of the perceiver’s leadership prototype (Epitropaki and Martin, 2005).

The next natural step in ILTs research was to identify the characteristics of this leadership prototype. From Lord et al. (1984) to the more recent works (e.g., Schyns and Schilling, 2011), a large number of attributes have been found to be associated with the leader category. In our study, we use the framework proposed by Epitropaki and Martin (2004), derived from the work of Offermann et al. (1994). Epitropaki and Martin (2004) focused on increasing the generalizability of individuals’ ILTs by using various employee groups as respondents. The eight original factors proposed by Offermann et al. (1994) were reduced to six, four of which related to the *leader prototype* and two of which related to the *leader anti-prototype*. *Intelligence, sensitivity, dedication*, and *dynamism* represent the prototypical characteristics of a leader; whereas *masculinity* and *tyranny* represent anti-prototypical factors. The factor structure proposed by Epitropaki and Martin (2004) has shown to be consistent over time and has presented little variance among employee groups.

A more recent development in Leadership Categorization Theory—the connectionist approach (e.g., Hanges et al., 2000; Brown and Lord, 2001; Lord and Shondrick, 2011)—has been devoting great attention to the contextual factors encompassing followers’ perceptual processes. More specifically, it introduces the concept of leadership schema or prototype activation. Depending on contextual factors such as the culture, perceivers’ demographic characteristics, leader gender, and the task’s nature, distinct leadership schemas will be activated by followers. Such a model allows individuals’ leadership prototypes to be fluid and contextually sensitive while maintaining their coherence and consistency (Lord et al., 2001).

However, despite the remarkable theoretical and empirical developments in the field of ILTs, areas remain to be advanced (see Foti et al., 2017). For instance, no experimental evidence shows that the ILTs factors proposed in previous models affect individuals’ *perceptions of leadership*. We consider that an experimental approach in this field can help test the causal effect of these attributes. Previous studies testing the effects of leader prototypicality (e.g., Engle and Lord, 1997; Epitropaki and Martin, 2005; Coyle and Foti, 2015; Riggs and Porter, 2017) have implicitly assumed that ILTs factors are equally important for explaining how individuals recognize someone as a leader. As such, these models do not allow each individual attribute’s importance to be assessed. Our study addresses this gap by showing how the recognition of each prototypical attribute influences followers’ leadership perceptions.

To address these issues, we conducted a series of three experimental studies using CA. In Study 1 we measure the relative importance of each of the six ILTs factors proposed by Epitropaki and Martin (2004) to test their contribution to the formation of leadership perceptions. For a more comprehensive view of this perceptual process, in Study 2 we explore the configural nature of leadership perceptions formation by testing interactions between ILTs factors. Finally, in Study 3 we test the variability of ILTs factors’ importance across contexts (e.g., business, military, religious, and political) to show ILTs’ dynamic nature. Before we describe the studies, we briefly present the CA methodology and show how it fits our research purpose.

## Conjoint Analysis Methodology

Conjoint analysis is an experimental design type in which a (usually large) set of factors is jointly manipulated. The method is commonly used in marketing research to determine the importance consumers assign to each of a product’s characteristics (e.g., color, shape, size, and brand), but it has also been applied in many other areas of social sciences (Rao, 2014). Standard experimental designs tend to focus on a small set of factors and sometimes do not allow researchers to estimate which of the components’ manipulation produce the observed outcome (Hainmueller et al., 2014). For example, in the field of leadership categorization, Cronshaw and Lord (1987) used vignettes to study the effects of a leader’s prototypicality, comparing experimental manipulations of prototypical and anti-prototypical leaders. Although their design allowed a test of the aggregate effects of leader prototypicality, it was unable to estimate the stimulus leader’s specific attribute effects. CA allows us to do precisely this type of examination.

In addition to the mentioned design distinction (use of a higher number of factors), another fundamental difference between CA’s statistical analysis and more typical experimental studies is the focus not only on the significance of each factor’s effect but also on its effect size and relative effect size. An attribute’s effect size scaled to percentage is interpreted as that factor’s importance (the sum of all factors’ importance reaches 100%) and offers a more intuitive measure of its relevance (Rao, 2014).

The use of CA has numerous advantages compared to simple importance ratings. First, because the respondent must rate not the attributes themselves but a set of profiles formed by a combination of attributes at different levels, problems of social desirability are mitigated (Ones and Viswesvaran, 1999; Wallander, 2009). A recent work by Tomassetti et al. (2016) demonstrates that CA (described as *policy capturing*) is more resistant to socially desirable responses than any of the commonly used self-reporting techniques, such as Likert-type choices, forced choices, ranking, and point-distribution techniques. Second, CA is more comparable to real-world decisions because choices are made regarding the overall situation not isolated attributes (Karren and Barringer, 2002). Finally, by manipulating cues and creating an orthogonal design, CA avoids the multicollinearity problems that are common in field data (Karren and Barringer, 2002).

We acknowledge that Soutar and Ridley (2008) applied CA to investigate the importance of specific leader *behaviors* from followers’ perspectives. However, they did not draw on any ILTs model, nor have they focused on leadership perceptions formation, given that the dependent variable they used was participants’ willingness to work with a hypothetical leader.

## Study 1—Heterogeneity of ILTs Factors’ Effects

Study 1 experimentally explores the formation of leadership perceptions based on the recognition of the 6 ILTs factors informed by Epitropaki and Martin (2004). We use CA to measure each factor’s importance and test whether they differ in terms of importance. As mentioned previously, this is an important test, because previous studies using Epitropaki and Martin’s (2004) model (e.g., Epitropaki and Martin, 2005; Coyle and Foti, 2015; Riggs and Porter, 2017) have focused on the effects of leader prototypicality assuming that all factors are equally important to this end. Moreover, these correlational studies did not focus on understanding how these leadership perceptions are formed. As such, little can be said about followers’ *information processing*, which is the basis of the socio-cognitive approach to leadership (Lord and Maher, 1991). We address these issues in the present study.

We assume that followers’ recognition-based leadership perceptions (Lord and Maher, 1991) result from the integration of the perceived level of a leader’s attributes (primarily those that compose followers’ ILTs), following, in general terms, the principles of Information Integration Theory (see Anderson, 1981, 2008). This theory explains how perceivers cognitively integrate an object’s many characteristics (e.g., the traits of a target person) to reach an overall impression. In this process, the weight given to each attribute by perceivers is usually not the same. These weights represent the importance, or the value, of a specific attribute (Anderson, 1971, 1981; Fiske, 1980), and these are exactly the attributes CA allows us to measure. Then, when followers integrate a leader’s many attributes to form an overall impression, some attributes will naturally receive more weight than others. We understand that it occurs because some attributes are more prototypical of the leader category, while others are less prototypical (see Lord et al., 1984). Therefore, we hypothesize as follows:

H1: *ILTs factors’ effects on leadership perceptions are heterogeneous, such that some attributes are more relevant than others for recognizing someone as a leader*.

Moreover, because the ILTs model in our experiments has prototypical and anti-prototypical attributes, we expect that the prototypical ones will positively affect one’s leadership perceptions, while the anti-prototypical attributes will have a negative effect.

### Methods

#### CA Design

The six factors validated by Epitropaki and Martin (2004) were used to create the leader descriptions. These factors were given two levels each (high and low). We did not use more than two levels for each attribute, because doing so would make the total number of combinations too large for a single respondent to rate, possibly leading to fatigue. These attributes’ levels were orthogonally combined to form the eight descriptions presented to each respondent. To make the quote for each attribute more representative of its respective factor, items that comprised each factor in Epitropaki and Martin’s (2004) study were used. **Table 1** shows the attributes, their levels and the descriptions used to manipulate the leaders’ characteristics.

**Table 1:** Attributes, levels and descriptions used in the manipulations.

Factor	Level	Description
Sensitivity	High	Sensitive (understanding, helpful)
	Low	Insensitive (unhelpful, unsympathetic)
Dedication	High	Dedicated (hardworking, motivated)
	Low	Undedicated (not hardworking, unmotivated)
Masculinity	High	Shows strong masculine behavior
	Low	Shows normal masculine behavior
Intelligence	High	Intelligent (clever, knowledgeable)
	Low	Unintelligent (unclever, unwise)
Dynamism	High	Dynamic (energetic, strong)
	Low	Not dynamic (slow, lifeless)
Tyranny	High	Tyrannical (manipulative, domineering)
	Low	Not tyrannical (democratic, not manipulative)

To create a full factorial design (all possible combinations of the six attributes, two levels each), we would need 64 (2^6^) profiles. However, using a fractional orthogonal design (Rao, 2014), the minimum profile number needed to study each factor’s main effects is 8.

#### Participants and Procedures

The sample consisted of 106 US participants recruited through Amazon MTurk. This online platform has shown to provide data “as reliable as those obtained via traditional methods” (Buhrmester et al., 2011, p. 3). Because of the within-subjects design (each participant rated eight different profiles), this number of participants delivered a total of 848 observations. Participants were 54% male, with an average age of 38.8 years (*SD* = 11.87), and 57% of them reported having at least a college degree. Of participants, 98% had prior job experience, and 93% had a boss or supervisor at least once.

The incentives paid to participants followed the MTurk standard of $6.00 per hour. This pay level was the same in the two subsequent studies. All studies have been conducted in accordance with the recommendations of the Institutional Review Board at the Brazilian School of Public and Business Administration (protocol number: 04012016 – 1721PP). The participants were provided a term of informed consent explaining the study’s purpose, that their participation was anonymous, and that they could withdraw their participation at any time.

**FIGURE 1:**
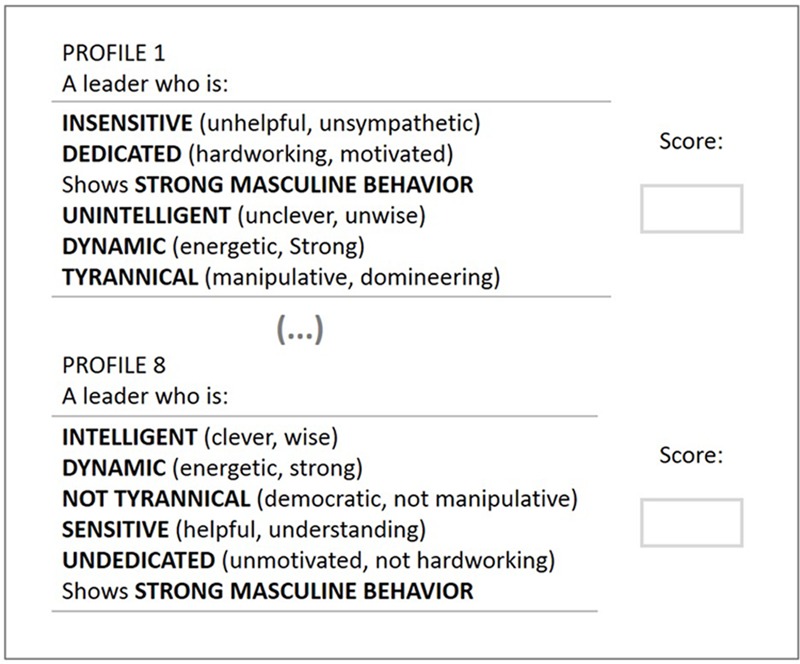
Example of the survey used in the studies. Each participant rated eight leader profiles.

Participants were told they would read descriptions of eight distinct leaders, each one presenting a different combination of six attributes. The attributes were described using the quotes as per **Table 1**. No further information about the leader type or context was provided. In this way, we activated a more generic leadership schema in respondents’ minds. We then measured the extent to which each leader profile approximated respondents’ mental representation of an effective leader. We did so by asking participants to rate each profile from 0 (not at all an effective leader) to 100 (a very effective leader). **Figure 1** shows how each participant was presented with the distinct leader profiles. Finally, all participants answered demographic questions.

### Results

The effects were estimated using a linear regression model with clustered standard errors because of the within-subjects design. The dependent variable was the score ascribed to each leader profile, and the independent variables were the attributes represented by a set of effects-coded variables [+1 (high), and -1 (low)]. In a model using effects-coded independent variables (+1 and -1), instead of dummy-coded variables (+1 and 0), the intercept (constant term) represents the grand mean, and the coefficients of each variable (*b*) are estimates of the deviations from the grand mean. The absolute values of attributes’ coefficients were used to calculate their relative importance.

Of the six ILTs factors, five presented significant effects. **Table 2** shows that among the prototypical attributes, *intelligence* (*b* = 10.30, *SE* = 0.72, *p* < 0.001), *sensitivity* (*b =* 9.03, *SE* = 0.62, *p* < 0.001) and *dedication* (*b* = 7.93, *SE* = 0.61, *p* < 0.001) are the most important. *Dynamism* (*b* = 2.75, *SE* = 0.62, *p* < 0.001) showed a remarkably lower importance in comparison with the other three prototypical attributes. *F*-tests show that dynamism’s effect is smaller than intelligence’s [*F*(1,105) = 67.52, *p* < 0.001], dedication’s [*F*(1,105) = 42.27, *p* < 0.001], and sensitivity’s [*F*(1,105) = 49.28, *p* < 0.001]. Among the anti-prototypical attributes, only *tyranny* had a significant effect on respondents’ perceptions (*b* = -1.61, *SE* = 0.69, *p* < 0.05). A leader’s *masculinity* was revealed to be irrelevant to respondents’ perceptions of leadership (*b* = -0.10, *SE* = 0.55, *p* = 0.85). Overall, these results support hypothesis 1 by showing a significant variance in ILTs factors’ importance. As expected, prototypical ILTs factors presented a positive effect and the anti-prototypical factors, negative effects, although for masculinity the effect was non-significant.

**Table 2:** Attributes’ effects and relative importance estimates.

	Perception of leadership		
Variables	*b*	*SE*	Relative importance
Intelligence	10.30***	0.72	32.46%
Sensitivity	9.03***	0.62	28.46%
Dedication	7.93***	0.61	24.99%
Dynamism	2.75***	0.62	8.67%
Masculinity	-0.10	0.55	0.32%
Tyranny	-1.62*	0.69	5.11%
Gender^1^	-6.76*	2.92	-
Age	-	0.11	-
Constant	37.26	4.66	-
*R*^2^	0.35		

*^*1*^Respondent gender: 0, male; 1, female. ^∗^p < 0.05, ^∗∗∗^p < 0.001.*

To further explore the non-significance of the masculinity factor, we tested its effects among demographic groups (male vs. female and younger vs. older respondents), to search for boundary conditions. Interaction analyses revealed that the masculinity factor’s effect did not differ (was equally non-significant) between female and male respondents [*b*_(gender__×__masculinity)_ = 0.69, *SE* = 1.79, *p* = 0.56]. However, it interacted with respondents’ age [*b*_(age__×__masculinity)_ = -0.09, *SE* = 0.04, *p* < 0.05], meaning that older respondents considered this dimension more negatively related to leadership than younger respondents. Nonetheless, this interaction’s effect size was rather small, so masculinity’s effect for those participants who were 1 *SD* above the mean (50.8 years old) remained non-significant (*b* = -1.51, *SE* = 0.93, *p* = 0.11).

### Discussion

Through this first study, we experimentally tested the effects of the ILTs factors proposed by Epitropaki and Martin (2004) on leadership impression formation. Our results show that a causal link exists between most of these factors and individuals’ leadership perceptions (only masculinity’s effect was shown to be non-significant). As predicted, and differently from previous empirical ILTs studies using congruence models have assumed (Engle and Lord, 1997; Topakas, 2011; Coyle and Foti, 2015; Riggs and Porter, 2017) remarkable variance exists in attributes’ importance in the leadership categorization process. Intelligence, dedication and sensitivity were shown to be the most meaningful attributes; whereas dynamism, tyranny and masculinity had lower importance. These findings may help to explain why Epitropaki and Martin (2005), and more recently Riggs and Porter (2017), did not find that the anti-prototype had significant effect in their studies. And, although Epitropaki and Martin (2005) and Riggs and Porter (2017) have found a significant effect of the prototypical dimension as a whole, because they aggregated the prototypical attributes in a single measure, the ILTs factors’ effects heterogeneity could not be revealed. However, when each of the ILTs factors’ effects is assessed individually, some attributes (e.g., intelligence) evidently explain a much greater variance of one’s leadership perceptions than others (e.g., dynamism).

Given that masculinity’s effect was shown to be non-significant, we explored this finding further. The idea that masculinity should be negatively related to prototypical leadership can be questioned. Indeed, some studies have demonstrated that perceptions of masculinity can be positively related to leadership emergence, leadership attitude (e.g., Kolb, 1999), and leadership stereotypes, although the link weakens over time (Koenig et al., 2011). Topakas (2011), for example, concludes that masculinity is an inappropriate dimension of the anti-prototype after assessments of the model’s goodness-of-fit. Additionally, we highlight that masculinity did not clearly emerge as a generalizable ILTs factor in the first factorial analysis performed in Offermann et al.’s (1994) study or in Schyns and Schilling’s (2011) content analysis. Therefore, we conclude that masculinity seems not to be an appropriate ILTs component, and we exclude this factor from our next analyses.

This first study challenged the assumption that all prototypical leader characteristics are equally meaningful for leadership categorization. By using CA, we can conclude that a causal link exists between the recognition of some (but not all) ILTs factors and *leadership perceptions*. However, Study 1 explores participants’ perceptions in a more simplistic or elemental way, i.e., only each attribute’s main effect was measured. Yet more advanced socio-cognitive theories propose that impression processes are configural in nature, in the sense that the perception of the whole is greater than the sum of the parts (Asch, 1946; Baumeister and Finkel, 2010; Fiske and Taylor, 2013). That is, the presence of some attributes can change the effect of other attributes. Specifically, Information Integration Theory suggests that this configurality can be assessed by testing the interaction of attributes with each other (Anderson, 2008). By performing these tests, we can show that attributes not only differ in terms of importance but can also be synergistically coupled with each other to predict leadership perceptions. To test these interactions, we move to Study 2.

## Study 2—Configural Nature of Leadership Perceptions

Social psychological research suggests that impressions are formed holistically, in the sense that the many attributes of a target object can interact with one another. As such, final impressions do not simply result from the sum of the perceived parts but from a more complex and interconnected perception of the whole (see Baumeister and Finkel, 2010; Fiske and Taylor, 2013). This perspective was first proposed by Asch’s (1946) configural model of people perception, which suggests that the importance of an object’s specific attribute is contingent on the presence of other attributes. Information Integration Theory also considers the configural nature of perceptions and proposes that it can be tested via interaction analysis (see Anderson, 2008). In our case, this configurality can be examined by testing the interaction effects of attributes with each other in the prediction of participants’ leadership perceptions. Foti and Hauenstein (2007) applied this more holistic perspective to predict individuals’ general leadership impressions (GLI) in a military setting. However, the focus of their study was not ILTs but variables such as self-efficacy, self-monitoring, and dominance.

The connectionist approach to leadership categorization (e.g., Lord and Shondrick, 2011) is also consistent with this perspective. This approach theorizes that followers may attribute a high level of prototypicality to a leader by recognizing a specific small set of prototypical attributes in him or her (Lord and Shondrick, 2011). When specific attributes are perceived in combination, the entire network of traits that compose one’s ILTs are activated, boosting attributions of leader prototypicality. In other words, specific attributes interact with each other in the formation of leadership perceptions, such that their combined effect is greater than the sum of their individual effects. Based on this holistic view of impression formation and on the connectionist approach to leadership categorization, we hypothesize as follows:

H2: *There will be interactions between ILT factors in predicting leadership perceptions*.

### Methods

#### Participants and Procedures

The sample included 150 participants. They were 59.3% female, with an average age of 38.37 (*SD* = 13.37), and 62% reported having at least a college degree. Of participants, 98% had prior job experience, and 96% had a boss or supervisor at least once. As in Study 1, participants were asked to evaluate different leader profiles in terms of perceived leadership effectiveness on a scale from 0 to 100. The ILTs factors described by Epitropaki and Martin (2004) were used to create the leader profiles, with the exception of the masculinity factor, for reasons set forth in Study 1’s “Discussion” section. In this study, both the main effects and the interactions are analyzed. Accordingly, a different research design is required. For an analysis of two-way interactions, we had to create 16 leader profiles in a fractional factorial design (see Rao, 2014). However, to avoid fatigue, each participant rated only one set of eight profiles. One of two sets of eight leader profiles was randomly assigned to each respondent. In each set of eight profiles (randomly ordered), the main effects are orthogonal; in the combination of both sets, all the two-way interactions are also orthogonal.

### Results

We tested for all possible two-way interactions between the 5 ILTs factors (a total of 10 combinations). Because by testing 10 different interactions increases we increased the likelihood of finding significant effects by chance, we reduced our significance level, considering as significant results only those with *p* < 0.001 that are robust across gender (male vs. female), age (older vs. younger, separated by the median age) and education level (completed college vs. otherwise). We consider *robust across conditions* interaction’s *p*-values smaller than 0.10 in each of these subgroups.

We found that the interactions of *dedication with intelligence* (*b* = 2.24, *SE* = 0.55, *p* < 0.001) and *dedication with sensitivity* (*b* = 2.57, *SE* = 0.63, *p* < 0.001) are highly significant, positive, and robust, according to our criteria. These interactions’ *p*-values were smaller than 0.05 in all subgroups, except for the interaction between dedication and sensitivity for the male group (*p* = 0.06). These results support hypothesis 2, showing that some ILTs factors significantly interact with others in the prediction of leadership perceptions. Additional results, which did not pass our criteria, are also presented in **Table 3**.

**Table 3:** Attributes’ effects, relative importance estimates and interactions.

	Perception of leadership				
	Model 1			Model 2	
Variable	*b*	*SE*	Relative importance	*b*	*SE*
Intelligence	9.20***	0.67	29.30%	9.20***	0.64
Sensitivity	6.28***	0.58	19.75%	6.28***	0.56
Dedication	8.65***	0.55	27.55%	8.65***	0.55
Dynamism	3.71***	0.42	11.81%	3.71***	0.42
Tyranny	-3.63***	0.53	11.56%	-3.63***	0.53
Gender^1^	-3.49	2.43		-3.49	2.43
Age	0.12	0.09		0.12	0.09
Dedication × Intelligence				2.24***	0.55
Dedication × Sensitivity				2.57***	0.63
Dedication × Dynamism				1.06*	0.52
Dedication × Tyranny				-0.85*	0.42
Constant	28.56	5.14		28.56	5.15
*R^2^*	0.32			0.35	
Δ*R*^2^				0.03*	

*N = 150. ^*1*^Respondent gender: 0, male; 1, female. ^∗^p < 0.05, ^∗∗∗^p < 0.001.*

### Discussion

The interactions of dedication with both intelligence and sensitivity confirmed our prediction that synergy between some of the ILTs factors would be observed. Specifically, these results mean that perceiving dedication along with either intelligence or sensitivity triggers an increased attribution of effective leadership, an attribution that goes beyond the simple sum of each factor’s effects. This finding is particularly important for ILTs theory, because prior studies did not account for these interactive effects. Our findings show that the additive assumption of most ILTs congruence models explains only part of the effect on followers’ perceptions. Significant interactions between attributes corroborate a more dynamic and holistic nature of leadership perceptions formation, in which an attribute’s importance is contingent on the presence of other attributes (Baumeister and Finkel, 2010; Fiske and Taylor, 2013). Although we recognize that the connectionist approach (e.g., Lord and Shondrick, 2011) has addressed this issue theoretically, empirical research has lagged behind (Foti et al., 2017).

These findings also provide extra support for hypothesis 1, and help deconstruct the assumption that all prototypical attributes are equally meaningful for leadership categorization. If some attributes can consistently enhance other attributes’ effects, while having their own direct effects, they should be considered as having a broader role in leadership categorization processes. They become almost necessary conditions to recognize someone as a leader.

Our findings thus far show that attributes’ importance is not homogeneous and is contingent on the presence of other attributes. However, all the results are limited to a general (an unspecified) leader type. Thus, we move on to Study 3 to examine how the activation of specific leadership schemas (e.g., military or business leadership) will influence the relative importance of ILTs factors.

## Study 3—The Contingency of Leader Attributes’ Importance

The connectionist approach to leadership categorization (e.g., Brown and Lord, 2001; Lord and Shondrick, 2011) argues that people’s leadership schema is context dependent. As such, the characteristics people expect leaders to have in a business environment may differ in some aspects from those expected from leaders in a military environment, for example. This difference occurs because different leadership schemas are automatically activated by contextual factors such as the task, the organization type, and the goals (Lord and Shondrick, 2011). Since the importance of an attribute for leadership categorization is expected to reflect how prototypical this attribute is in a specific context, we hypothesize as follows:

H2: *The effects of ILTs factors on leadership perceptions will be contingent on the leadership context*.

However, although leadership prototypes are expected to vary as a function of context, they are also expected to preserve some consistency and coherence (Lord et al., 2001). This expectation is because they are constrained by a superordinate leadership prototype people hold in mind—a more generic prototype of the leader category that guides all other basic-level prototypes (e.g., of a military, religious, or business leader; Lord et al., 1984). Based on this rationale, we believe that, despite the expected variability of ILTs factors’ importance across contexts, an overall pattern of attributes’ importance should be observed. Regardless of the context, the ordering of each attribute’s importance in recognizing someone as a leader will be relatively stable.

### Methods

#### Participants and Procedures

The sample included 310 participants, 53.3% of whom were female, with an average age of 38.54 years (*SD* = 12.86). Of respondents, 58.3% reported having at least a college degree. All participants had prior job experience, and 94% had a boss or supervisor at least once. The procedure was similar to those used in previous studies; the only difference was that respondents were randomly assigned to one of four leadership contexts (business, military, political, and religious). We chose these contexts because they were used by Lord et al. (1984) and are familiar to most participants. To activate these leadership prototypes in respondents’ minds, they were asked to write down two activities commonly performed by a leader in that context. Then, they rated each profile from 0 to 100, as in Studies 1 and 2. However, in this case, they knew that the leader was, for instance, a political leader (in Studies 1 and 2, no information about the leader type was provided). Finally, they answered demographic questions.

### Results

Interaction analysis (attributes × context) showed that intelligence’s effect is significantly higher for business leaders than for religious ones (*b* = 2.82, *SE* = 1.12, *p* < 0.05), and the tyranny’s effect was significantly more negative for religious leaders than for military (*b* = -4.15, *SE* = 1.13, *p* < 0.001), political (*b* = -2.30, *SE* = 1.7, *p* < 0.05), and business leaders (*b* = -3.04, *SE* = 1.13, *p* < 0.001). Interestingly, tyranny’s main effect was non-significant for military leaders (*b* = -0.61, *SE* = 0.73, *p* = 0.41). Moreover, sensitivity was significantly less important for military leaders than for religious (*b* = -2.96, *SE* = 1.09, *p* < 0.001) and business leaders (*b* = -1.97, *SE* = 0.83, *p* < 0.05). Lastly, we found that dedication is more important for business leaders than for religious leaders (*b* = 2.43, *SE* = 1.09, *p* < 0.05). Overall, these findings support hypothesis 3 by showing that ILTs factors’ importance significantly vary across contexts (**Table 4**).

**Table 4:** Attributes’ effects by leadership context.

	Perception of leadership by context			
Variable	Business (*b*)	Political (*b*)	Military (*b*)	Religious (*b*)
Intelligence	11.61^∗∗∗^	9.37^∗∗∗^	10.63^∗∗∗^	8.78^∗∗∗^
	>P^†^; >R^∗^	<B^†^		<B^∗^
Dedication	9.00^∗∗∗^	7.06^∗∗∗^	8.06^∗∗∗^	6.65^∗∗∗^
	>P^†^; >R^∗^	<B^†^		<B^∗^
Sensitivity	8.42^∗∗∗^	7.31^∗∗∗^	6.44^∗∗∗^	9.41^∗∗∗^
	>M^∗^		<B^∗^; <R^∗∗^	>M^∗∗^
Dynamism	3.35^∗∗∗^	2.56^∗∗∗^	3.85^∗∗∗^	1.86^∗^
			>R^†^	<M^†^
Tyranny	-1.71^∗^	-2.46^∗∗∗^	-0.61	-4.76^∗∗∗^
	>R^∗∗∗^	<M^†^; >R^∗^	>P^∗^; >R^∗∗∗^	<B^∗∗∗^; <P^∗^; <M^∗∗∗^

*B, business; P, political; M, military; R, religious. To keep the table clean, only coefficients are shown. ^†^p = 0.05, ^∗^p < 0.05, ^∗∗^p < 0.01, ^∗∗∗^p < 0.001.*

Although not the primary focus of this study, interaction analysis of attributes with respondents’ gender and age also revealed some significant results. The interactions between sensitivity and gender (*b* = 2.35, *SE* = 0.67, *p* < 0.001) and between sensitivity and age (*b* = 0.07, *SE* = 0.02, *p* < 0.001) were highly significant, meaning that both female and older respondents considered this attribute more important than their counterparts (these interaction terms were tested simultaneously in the same model).

### Discussion

Indeed, the activation of distinct leadership schemas caused participants to ascribe distinct weights to the same attributes. This finding provides empirical support to the schema activation hypothesis proposed in the connectionist approach to leadership categorization (e.g., Brown and Lord, 2001; Lord and Shondrick, 2011). So far, few studies have empirically tested this theoretical approach’s premises (e.g., Foti and Hauenstein, 2007). Importantly, the results also show that, despite the hypothesized variability, attributes’ relative importance conserved some consistency across contexts. Specifically, and confirming our previous studies’ results, intelligence, dedication and sensitivity were shown to be the most important attributes in all contexts, and the least important ones were dynamism and tyranny. We highlight the fact that tyranny seems not to be part of the leadership *anti*-prototype when a military leadership schema is activated in participants’ minds. That is, in a context related to high levels of discipline, warfare, and national defense, leaders have kind of a “license to tyrannize.”

We also found that sensitivity interacted with gender and age. Fiske et al. (2007) argue that, in social judgments, women give more weight than men to characteristics related to warmth. This pattern seems to replicate in the case of leadership perceptions, as our results suggest. In relation to age, because individuals’ ILTs are developed through followers’ past experiences with leaders (Epitropaki and Martin, 2004), in accumulating bad experiences with insensitive leaders (or good experiences with sensitive ones), people may learn about this attribute’s importance for leadership.

## General Discussion

Studies in the ILTs field using congruence models (Epitropaki and Martin, 2005; Topakas, 2011; Coyle and Foti, 2015; Riggs and Porter, 2017) have implicitly assumed and reinforced the idea that all the leader attributes proposed by Epitropaki and Martin’s (2004) model are equally important for the formation of leadership perceptions. Based on our results, we conclude that this assumption is not accurate, for the following reasons. First, on average, leader attributes’ effects significantly differ (e.g., dedication, intelligence, and sensitivity have been shown to be more important than dynamism, tyranny, and masculinity); second, people value attributes differently (e.g., female and older respondents, valued sensitivity more); third, attributes interact with each other (e.g., dedication interacted with both intelligence and sensitivity); and fourth, the context will partially dictate an attribute’s importance (e.g., in a military context, tyranny seems to be irrelevant).

Based on these results, we believe that field studies using leader prototypicality measures should engage in a more person-oriented approach, rather than a variable-oriented approach, as is usual. According to Foti et al. (2017), a person-oriented approach allows for inter-individual variation within a system of variables, instead of forcing participants to consider only a specific set of attributes informed by researchers.

Our results help to explain some inconsistent findings from previous studies. For instance, Topakas (2011) found that the masculinity factor did not contribute to her ILTs model goodness-of-fit. Accordingly, Epitropaki and Martin (2005) and Riggs and Porter (2017) did not find a significant effect of the anti-prototypical dimension, represented by both masculinity and tyranny. According to our experiments, these two factors have been shown to be less important for leadership categorization, which implies that they are not major ILTs factors. Thus, these seemingly inconsistent findings from previous studies are, in fact, consistent.

We have provided support to the notion that leadership perceptions do not simply result from an additive integration of a stimulus person’s characteristics, as previous research has assumed. The formation of leadership perceptions is a more holistic process, in which the whole is not only the sum of the parts. To our view, the existence of highly significant interactions between attributes suggests that ILTs can be somehow divided into two categories: necessary and complementary attributes. In other words, if some necessary attributes are not present, other attributes, although prototypical, will have a limited effect on leadership perceptions. Dedication seems to be one of these necessary leader attributes.

Considering all experiments, we have presented empirical evidence that a clear causal link exists between the recognition of some attributes (intelligence, dedication, and sensitivity) and leadership perceptions. Demonstrating this causal link is another of our work’s contributions. This experimental study is the first to test the effect of early proposed attributes on individuals’ leadership perceptions. It seems to be a contradiction, since the formation of leadership perceptions is the core of the socio-cognitive approach to leadership. However, to date, researchers have only asked participants which attributes they believe are important for leaders to have or to rate a list of traits and behaviors (e.g., Offermann et al., 1994). Astonishingly, we could not find any experimental study testing if these attributes have, in fact, an effect on leadership perceptions. Our research contributes to fill this gap.

From a methodological perspective, CA has been shown to be a quite valuable tool for ILTs research, because it can capture the importance ascribed by perceivers to each of a target person’s attributes. Because each attribute is presented to participants in the context of all other attributes, CA’s experimental design approximates more real-world situations (Karren and Barringer, 2002). Moreover, CA presents advantages when compared to common self-reporting methods, such as resistance to socially desirable responses (Tomassetti et al., 2016) and the mitigation of multicollinearity problems, both of which are common in field data (Karren and Barringer, 2002).

We believe that our results are also relevant for leadership practice. By showing which attributes impact followers’ leadership perceptions most and their consequent attitudes and behaviors, we can inform leaders about which attributes to develop, or, at least, about which attributes to make more salient in first contacts with followers. In this matter, Engle and Lord (1997, p. 991) emphasize the importance of a leader making a good first impression on subordinates: “once a perceiver has labeled another individual (the leader), it is difficult to change that initial impression.” We highlight the fact that female and older participants valued sensitivity more than their counterparts. Regarding female respondents, our findings corroborate previous leadership research (Deal and Stevenson, 1998; Epitropaki and Martin, 2004) and align with socio-cognitive theories on impression formation (Fiske et al., 2007). Thus, leaders must be aware of the fact that their female subordinates’ attitudes will be strongly affected by their level of consideration and warmth in the leader-follower relationship. Although the same pattern has been observed in older participants, we believe that more evidence is needed to convert this finding into a recommendation for practitioners. Another important point to be considered by real-world leaders is the interaction effect of dedication with all other attributes (especially with intelligence and sensitivity). That is, beyond its own inherent importance for a follower’s leadership perception, dedication strengthens (or the lack thereof weakens) the effects of other important attributes. In other words, dedication seems to be a necessary condition for someone to be perceived as a highly effective leader.

## Limitations and Future Research

Although we consider that the ILTs model proposed by Epitropaki and Martin (2004) was useful for our study, we also believe that it may impose several limitations on our conclusions. Followers in real-world situations may consider attributes other than those proposed by this model. Thus, future works can either follow a more person-oriented approach (Foti et al., 2017) or focus on attributes proposed by different ILTs models (e.g., Gerstner and Day, 1994; Offermann et al., 1994; Den Hartog et al., 1999; Ling et al., 2000; Schyns and Schilling, 2011).

Finally, based on our findings, we believe that future empirical research should consider the fact that (i) leader attributes are not equally meaningful, (ii) the context can significantly influence the meaning of attributes, and (iii) they interact with each other. These findings indicate that, to assess a leader’s prototypicality, researchers should avoid using congruence measures based on an unweighted summation of differences between the ideal and the perceived level of specific leader attributes. Instead, they could adopt at least three other strategies to measure a leader’s prototypicality: first, if using aggregate congruence measures (following either a person- or a variable-oriented approach), researchers could weigh each attribute by its relative importance, which can be either informed by participants directly or measured with CA; second, by using Venn diagrams through which participants could inform the extent to which their leaders match their idealized image of a leader (see Van Quaquebeke et al., 2014); and third, by using scales such as the GLI (Cronshaw and Lord, 1987). However, we believe that accounting for the interaction between attributes would not be feasible with congruence measures, since the pattern of interactions may vary from person to person. Thus, the use of followers’ overall leadership impressions (through Venn diagrams or scales) may be the best solution to measure a leaders’ prototypicality.

## Ethics Statement

This study was carried out in accordance with the recommendations of the ProPesquisa Institutional Review Board at the Brazilian School of Public and Business Administration with informed consent from all subjects. All subjects were provided a term of informed consent in accordance with the Declaration of Helsinki. The protocol was approved by the Institutional Review Board (protocol number: 04012016 - 1721PP).

## Author Contributions

FA, FS, RG, and GT developed the idea of applying conjoint analysis in the ILTs field. GT and FS structured the theoretical background. RG and GT structured the experiments. GT ran the experiments. GT and RG did the analyses. GT and RG wrote the methodology section (conjoint analysis). GT, FS, and FA wrote all of the other sections.

## Conflict of Interest Statement

The authors declare that the research was conducted in the absence of any commercial or financial relationships that could be construed as a potential conflict of interest.
